# Age-dependent cognitive impairment, hydrocephalus and leukocyte infiltration in transgenic mice with endothelial expression of human EPHX2

**DOI:** 10.1038/s41514-022-00090-1

**Published:** 2022-07-05

**Authors:** Catherine M. Davis, Wenri H. Zhang, Thierno M. Bah, Natalie E. Roese, Elyse M. Allen, Philberta Leung, Sydney J. Boutros, Tessa Marzulla, Esha Patel, Xiao Nie, Farah N. Alkayed, Justin H. Huang, Michael A. Jensen, Jacob Raber, Martin M. Pike, Nabil J. Alkayed

**Affiliations:** 1 Department of Anesthesiology & Perioperative Medicine, Oregon Health & Science University, Portland, OR 97239 USA; 2 Department of Behavioral Neuroscience, Oregon Health & Science University, Portland, OR 97239 USA; 3 Departments of Neurology and Radiation Medicine, Division of Neuroscience, ONPRC, Oregon Health & Science University, Portland, OR 97239 USA; 4 Advanced Imaging Research Center, Oregon Health & Science University, Portland, OR 97239 USA; 5 Knight Cardiovascular Institute, Oregon Health & Science University, Portland, OR 97239 USA

**Keywords:** Cognitive ageing, Learning and memory

## Abstract

Soluble epoxide hydrolase (sEH) is upregulated in microvascular endothelium of human brain with vascular cognitive impairment (VCI). Transgenic endothelial expression of human sEH in mice (Tie2hsEH) induces endothelial dysfunction (ED), a pathogenetic mechanism of VCI. We sought to determine if endothelial upregulation of sEH is sufficient to cause cognitive impairment, and if cognitive impairment due to chronic hypoperfusion induced by unilateral common carotid artery occlusion (CCAO) is exacerbated in Tie2hsEH mice. Behavioral performance was assessed by the open field, rotarod, novel object, Morris water maze and fear conditioning tests. Cerebral blood flow and brain morphology were evaluated by MRI, and inflammatory changes investigated using immunohistochemistry and flow cytometry. We demonstrate that transgenic endothelial expression of sEH is sufficient to induce cognitive impairment, associated with leukocyte infiltration, brain atrophy and accelerated, age-dependent ventriculomegaly, identifying ED and sEH upregulation as potential underlying mechanisms and therapeutic targets for VCI.

## Introduction

Vascular cognitive impairment (VCI) is the second most common cause of dementia following Alzheimer’s disease (AD). Historically considered distinct, VCI- and AD-related dementias often co-exist and exacerbate each other, emphasizing the vascular contributions to dementia^1,2^. Although the pathogenesis of VCI is not fully understood, small vessel disease is a key underlying factor, brought about in part by dysfunctional cerebrovascular endothelium^3^.

Cytochrome P450 (CYP) derivatives of arachidonic acid are important regulators of the cerebral microcirculation. Specifically, 14,15-epoxyeicosatrienoate (14,15-EET) plays important physiological and pathophysiological functions in brain, including regulation of cerebral blood flow (CBF) and protection against ischemic and inflammatory brain injury^4^. The actions of 14,15-EET are terminated upon conversion to the less active metabolite, 14,15-dihydroxyeicosatrienoate (14,15-DHET) by the enzyme soluble epoxide hydrolase (sEH), which is encoded by the *ephx2* gene. sEH is upregulated in mouse brain microvessels by chronic high fat diet, concomitant with cognitive impairments^5,6^. We also observed increased sEH expression in microvascular endothelium of postmortem human brain tissue with a history of dementia and small vessel disease, compared to age- and sex-matched controls^7^. It is not clear, however, if endothelial sEH upregulation in mice and humans is causally linked to cognitive impairments, or a mere association. Transgenic upregulation of human sEH in mice impairs endothelial-dependent vasodilation^8^ and attenuates the functional hyperemic response to whisker stimulation^9^. However, it is not known whether endothelial dysfunction in these mice is sufficient to induce cognitive impairments, and if impaired cerebral blood flow regulation is the underlying mechanism. Therefore, in the current study, we determined if transgenic upregulation of human sEH in endothelium is sufficient to cause cognitive impairments. A secondary goal was to determine if transgenic endothelial sEH upregulation exacerbates cognitive impairment due to chronic hypoperfusion induced by unilateral common carotid artery occlusion (CCAO)^10^.

## Results

To determine if transgenic expression of endothelial sEH is sufficient to induce cognitive impairment, as well as whether it exacerbates hypoperfusion-induced cognitive impairment, 3-month-old male WT and Tie2hsEH mice were subjected to CCAO or sham surgery, and assessed for neurobehavioral, CBF and histological outcomes 3–4 months later^10^. As males are at higher risk of developing VCI^11,12^, male mice were used in this study. Levels of EETs and DHETs were measured in plasma and cerebral endothelial cells acutely isolated from WT and Tie2hsEH mice.

### DHETs are increased in endothelial cells from Tie2hsEH and CCAO mice; plasma EETs are decreased

**Fig. 1:**
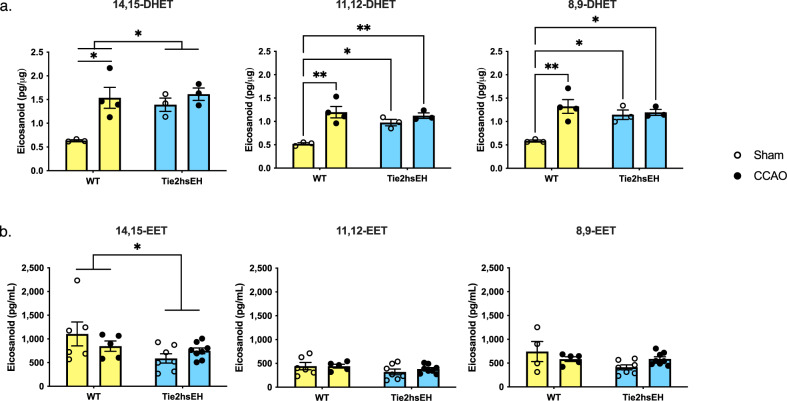
Endothelial DHETs are increased in Tie2hsEH mice and by CCAO, plasma EETs are decreased. Four months following surgery DHETs were assessed in acutely isolated cerebral endothelial cells (**a**) and EETs in plasma (**b**) by LC–MS/MS. **a** Endothelial 14,15-DHETs were increased in Tie2hEH mice compared to WT, 11,12- and 8,9-DHETs are also increased in Tie2hsEH sham endothelium compared to WT sham; 14,15-, 11,12- and 8,9-DHET were all increased by CCAO surgery in WT but not Tie2hsEH mice (*n* = 3–4/group). **b** Plasma 14,15-EETs were reduced in Tie2hsEH mice compared to WT; there was no effect of surgery. *p < 0.05, **p < 0.01, 2-way ANOVA, *n* = 4–8/group. Bar graphs display mean ± SEM.

To confirm that transgenic expression of human sEH increases EETs metabolic conversion to DHETs, we first assessed whether levels of DHETs were increased in endothelial cells isolated from 7-month-old Tie2hsEH mice compared to age-matched WT mice, and in mice of both genotypes subjected to CCAO (Fig. 1a). Four months following CCAO, DHETs in acutely isolated cerebrovascular endothelial cells were analyzed by LC–MS/MS. sEH products of all detectable EETs regio-isomers (14,15-, 11,12- and 8,9-DHET) were increased in Tie2hsEH compared to WT mice (*p* < 0.04, *n* = 3–4 mice/genotype). In WT mice, DHET concentrations following CCAO surgery increased (14,15-, 11,12- and 8,9-DHET concentrations were all increased by CCAO compared to sham surgery: *p* < 0.02, *n* = 3–4 mice/genotype) to a level comparable to that seen in sham-surgery Tie2hsEH mice. These data suggest that CCAO induces endothelial expression of sEH, hinting at a common mechanism between the two models (Tie2hsEH and CCAO). In contrast to WT mice, CCAO surgery had no additional effect on DHET levels in Tie2hsEH mice.

We next determined if endothelial expression of human sEH or CCAO surgery alters circulating EETs levels (Fig. 1b). Endothelial sEH expression selectively reduced plasma 14,15-EET levels, which were reduced from an average of 976.85 ± 129.31 pg/mL in WT mice to 669.02 ± 80.13 pg/mL in Tie2hsEH mice (*F* (1,22) 4.762, *p* = 0.04), with no effect on either 11,12- or 8,9-EET. Surgery had no effect on plasma EET levels in either genotype (*n* = 4–8/ genotype).

### Increased endothelial sEH expression and CCAO lead to impaired spatial memory retention

**Fig. 2:**
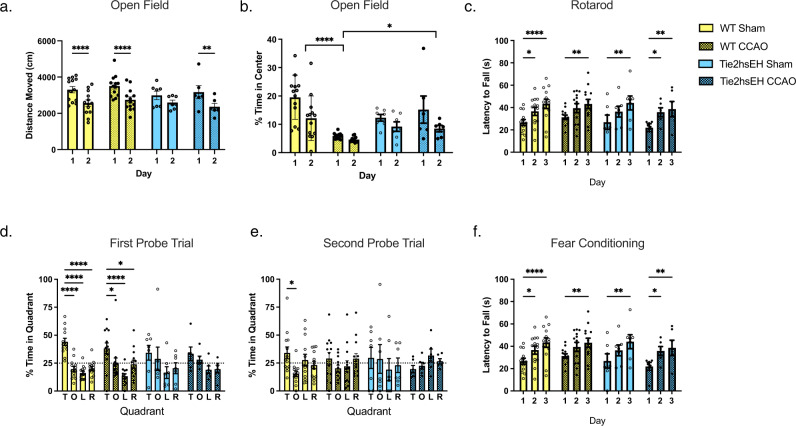
Tie2hsEH genotype and CCAO lead to impaired spatial memory retention. Three months after surgery behavioral performance was assessed. Locomotor activity and anxiety- related behavior were assessed by the open field test. **a** Locomotor activity was unaffected by genotype or surgery. WT sham, WT CCAO and Tie2hsEH UCCAO groups all exhibited reduced distance moved on day 2 of testing. **p < 0.01, ****p < 0.0001 vs. day 1 of the same group, 2-way ANOVA with Sidak’s multiple comparisons test, *n* = 6–13/group. **b** Anxiety-related behavior was determed by time spent in the more anxiety-provoking center of the open field; time spent in the center was reduced by CCAO compared to sham in WT. Time in center was unaffected by genotype in sham, or by CCAO in Tie2hsEH, compared to their sham. *p < 0.05, ****p < 0.0001, 2-way ANOVA with Tukey’s multiple comparisons test, *n* = 7–13/group. **c** Locomotor performance was assessed by rotarod test. All groups, regardless of surgery or genotype improved performance over the 3 days of testing; no differences were detected between groups. *p < 0.05, **p < 0.01, ****p < 0.0001 vs. trial 1 of the same group, 2-way ANOVA with Tukey’s multiple comparison test. Spatial learning and memory were assessed in the Morris water maze test. **d** In the first probe trial, WT sham and CCAO mice spend more time in the target quadrant than the other quadrants. Tie2hsEH mice, both sham and CCAO, did not show preference for a particular quadrant. **e** In the second probe trial, WT sham mice spend more time in the target compared to the opposite quadrant, however the WT CCAO group did not. In the second probe trial, neither Tie2hsEH sham nor CCAO showed preference for a quadrant. T target, O opposite, L left, R right. *p < 0.05, ****p < 0.0001, 2-way ANOVA with Tukey’s multiple comparison test, *n* = 6–13/treatment. **f** No differences between groups were observed in contextual fear memory tasks, 2-way ANOVA, *n* = 6–13 mice/genotype/treatment. Bar graphs display mean ± SEM.

Three months after CCAO, behavioral performance was assessed, when mice were 6 months old. First, locomotor performance and anxiety-related behavior were assessed using the open field and rotarod tests. The open field test revealed no differences in locomotor activity (distance moved) between experimental groups (Fig. 2a). Anxiety-related behavior was assessed by determining time spent in the more anxiety-provoking center of the open field (Fig. 2b); we found that surgery had an effect on WT anxiety levels, with WT CCAO mice spending less time in the center (*p* < 0.0001, 2-way ANOVA), however there were no genotype differences, nor was there an effect of CCAO in Tie2hsEH compared to their shams, there was also genotype × surgery interaction (*F*(1, 70) 17.19, *p* < 0.0001). These results indicate that CCAO induces anxiety-related behavior in WT mice, however neither genotype nor surgery do so in Tie2hsEH mice. Locomotor performance assessed by rotarod, this was also unaffected by either surgery or genotype (Fig. 2c); all groups improved their performance with training over the 3 days of testing. The novel object memory test was carried out to assess non-spatial memory. Tie2hsEH mice showed intact object recognition, and performance was not affected by CCAO; both groups showed a preference for the novel object compared to familiar (57.83 ± 5.00% and 61.60 ± 7.09% time exploring the novel object, respectively; Supplementary Fig. 1). Next, in the water maze, there were no genotype differences in ability to learn the visible or hidden platform location in sham or CCAO mice. However, transgenic endothelial expression of human sEH impaired spatial memory retention, as assessed in the water maze probe trial (no platform). In the first probe trial, 24 h after the last hidden platform training trial (Fig. 2d), WT mice, both sham and CCAO, spent more time in the target quadrant than in any other quadrants (44.05 ± 3.26% and 38.26 ± 4.91%; *p* < 0.05, 2-way ANOVA, respectively), indicating intact spatial memory retention. However, this spatial bias for the target quadrant was absent in Tie2hsEH mice, both in sham and CCAO Tie2hsEH mice (*n* = 6–13/treatment), indicating impaired spatial memory retention. In the second probe trial, 48 h after the first probe trial (Fig. 2e), WT sham mice still spent more time in the target quadrant than the opposite quadrant; however, this significance was lost in WT mice subjected to CCAO. In the second probe trial, neither sham nor CCAO Tie2hsEH mice showed a spatial bias for the target quadrant (*n* = 6–13/treatment). No differences were observed in contextual fear learning or memory tasks between genotypes or treatments (Fig. 2f). Taken together, these results suggest that Tie2hsEH mice are able to learn; however, they have impaired spatial memory retention that is more pronounced than in WT mice after CCAO. This memory deficit is specific to spatial memory, as hippocampus-dependent contextual fear memory was unaltered, as was non-spatial memory, and was also not due to potential genotype differences in swim speeds, locomotor impairments, or anxiety.

### Hippocampal blood flow is reduced by CCAO, but not by transgenic sEH expression

**Fig. 3:**
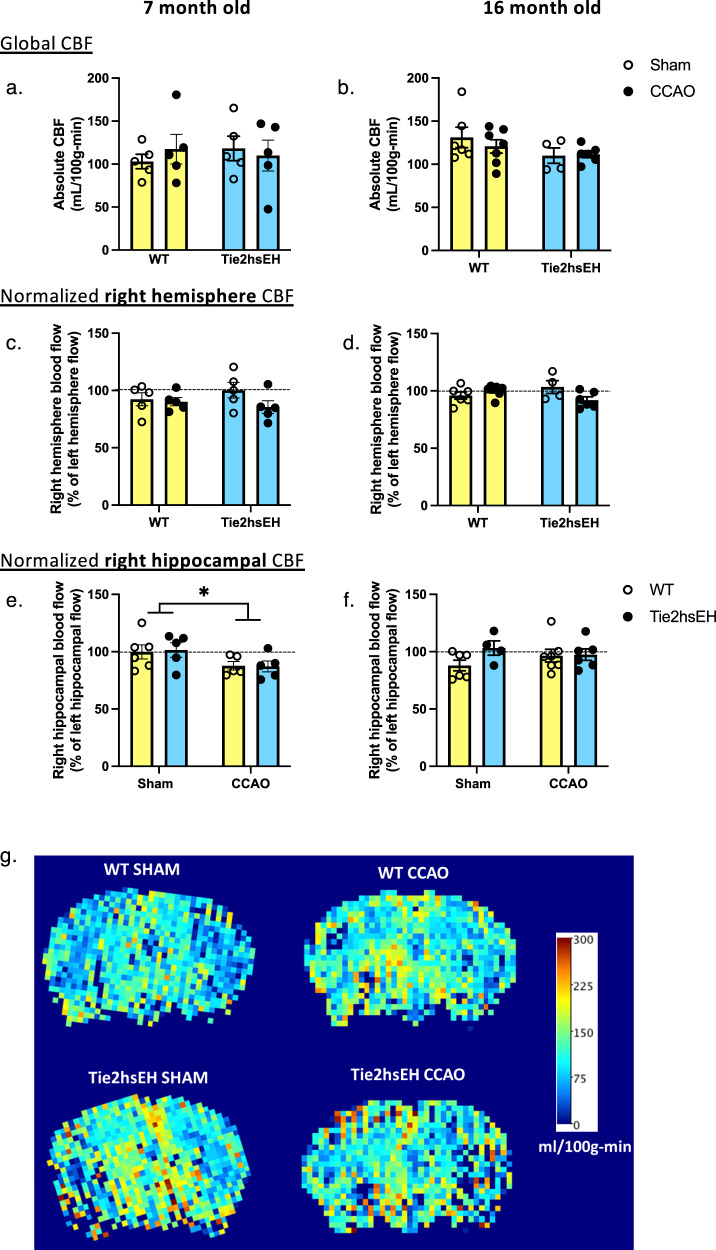
Unilateral hippocampal decrease in CBF in CCAO mice. CBF was measured using ASL-MRI perfusion in 7- and 16-month-old mice, 4 months following CCAO surgery. Global CBF, and right hemispheric CBF, were unaffected by surgery in either genotype (**a**–**d**). Hippocampal blood flow in the ipsilateral hemisphere was reduced in both WT and Tie2hsEH 7-month-old, but not 16-month-old mice (**e**, **f**). **g** Representative CBF maps from 7-month-old cohort. *p < 0.05, 2-way ANOVA, *n* = 4–6/group, data are represented as mean ± SEM.

To determine if cognitive impairments in Tie2hsEH mice are associated with chronic hypoperfusion, as a potential mechanism, CBF was assessed by ASL-MRI. Furthermore, to determine if there is an age-dependent effect on hypoperfusion, CBF was measured in young and old. The younger cohort (following behavioral testing) underwent sham or CCAO surgery at 3 months of age, and the older mice at 12 months of age. Both cohorts were assessed 4 months following CCAO; at 7 and 16 months of age, respectively. Neither genotype nor surgery affected global CBF at either age (Fig. 3a, b). When blood flow in the ipsilateral hemisphere was normalized to the contralateral hemisphere, no differences were observed in response to either surgery or genotype (Fig. 3c, d).

Since the spatial learning memory retention in the water maze probe trial is hippocampus-dependent, hippocampal blood flow was also measured. In the younger cohort, we observed a surgery-associated decrease in hippocampal CBF in the ipsilateral hemisphere; this was true in both WT and Tie2hsEH genotypes (*F* (1, 17) 5.835, *p* = 0.03; Fig. 3e). When normalized to the contralateral hemisphere, blood flow was reduced from 99.83 ± 6.13% to 87.70 ± 3.69% in the WT ipsilateral hemisphere. Similarly, in Tie2hsEH mice flow was reduced from 101.50 ± 6.36% to 87.18 ± 4.74%. No effect of either surgery or genotype was observed in the 16-month-old cohort (Fig. 3f). This result suggests that reduced hippocampal blood flow may contribute to the CCAO-induced cognitive impairment in WT mice (Fig. 2d), but not to the genotype-specific cognitive impairment observed in Tie2hsEH mice.

### Hippocampal size and synaptic density are unaltered in Tie2hsEH mice, or after CCAO

To further examine morphological changes in hippocampus as a potential mechanism for the cognitive deficits observed in Tie2hsEH mice, hippocampal volume was assessed by T_2_-weighted MRI, and hippocampal synapse density by immunoreactivity of the synaptic marker synaptophysin (Supplementary Fig. 2). No differences were observed in hippocampal volume in response to surgery in either genotype (Supplementary Fig. 2a, b). Synaptophysin immunoreactivity was assessed in the younger cohort; no changes in synaptophysin immunoreactivity were observed in CA1, CA3 or dentate gyrus regions of the hippocampus in Tie2hsEH or in WT mice after CCAO (Supplementary Fig. 2c–e). These data indicate that the cognitive impairments observed in the Tie2hsEH mice are not due to gross morphological hippocampal changes.

### Reduced brain volume and ventriculomegaly in Tie2hsEH mice

**Fig. 4:**
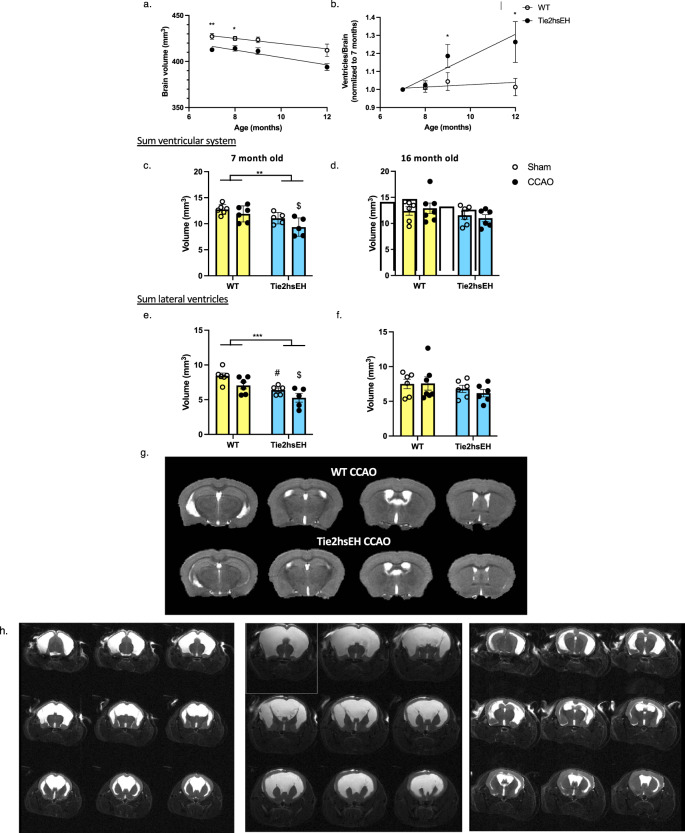
Age-dependent atrophy and ventriculomegaly in Tie2hsEH mice. Ventricular and total brain volume were measured by T_2_-weighted MRI. Longitudinal T_2_-weighted MRI shows that Tie2hsEH mice have a smaller brain volume than WT at 7 and 8 months of age (**a**), *p < 0.05, **p < 0.01, 2-way ANOVA with Sidak’s multiple comparison test, *n* = 4–18/group. Ventricle size, normalized to brain volume, increases with age in Tie2hsEH mice, but not WT (**b**); linear regression analysis shows that the slope of Tie2hsEH is steeper than that of WT (0.06 vs. 0.006, respectively, *p* = 0.0038), with increased ventricle/brain ratio in Tie2hsEH mice at 9 and 12 months of age, *p < 0.05, 2-way ANOVA with Sidak’s multiple comparison test, *n* = 3–14/group. Ventricular volume is reduced in Tie2hsEH mice compared to WT, in 7-month-old mice (**c**), but not in 16-month-old-mice (**d**); CCAO surgery does not alter ventricle volume in either age group. Lateral ventricles are also reduced in volume in the 7- but not 16-month cohort (**e**, **f**); a reduction in volume is also observed by CCAO surgery in the 7- but not 16-month cohort. *p < 0.05, ***p < 0.001, #*p* < 0.05 WT sham vs. Tie2hsEH sham, $*p* < 0.05 WT CCAO vs. Tie2hsEH CCAO, 2-way ANOVA with Sidak’s multiple comparison test, *n* = 5–7/group. Representative sequential *T*_2_-weighted image slices are shown of both genotypes at 7 months of age (**g**). Severe hydrocephalus was observed in 3 out of 18 naïve12-month-old Tie2hsEH mice, but not WT, by ASL-MRI perfusion (n/s Fisher’s exact test); MRI images of the 3 hydrocephalic Tie2hsEH brains (**h**). Data are represented as mean ± SEM.

Because aging and cognitive impairments are associated with brain atrophy^13^, we measured brain volume in WT and Tie2hsEH mice. Longitudinal studies of naïve mice revealed that brain volume decreased with age, regardless of genotype (*F* (2.17, 35.38) = 23.89, *p* < 0.0001), with a smaller brain volume in Tie2hsEH mice compared to WT mice (*F* (1,31) 13.53, *p* = 0.001), with Sidak’s post-hoc analysis multiple comparisons test showing significant differences at 7 and 8 months of age (*p* < 0.05, *n* = 4–18/genotype/age; Fig. 4a). Linear regression analysis determined that there was no difference in slopes between genotypes (*F* (1, 84) 0.66, *p* = 0.42), indicating that age-dependent brain atrophy occurs at the same rate in both genotypes, however Tie2hsEH mice have a smaller brain volume throughout.

Since expansion of lateral ventricles is clinically associated with vascular dementia^14^, we carried out longitudinal *T*_2_-weighted MRI studies of ventricular size in naïve mice of both genotypes (WT and Tie2hsEH), from 7- to 12-months of age (Fig. 4b). When normalized to brain volume, ventricle size was significantly larger in Tie2hsEH than WT mice (*F* (1, 22) 9.16, *p* = 0.006), with Sidak’s post-hoc analysis multiple comparisons test showing significant differences at 9 and 12 months of age (*p* < 0.05); there was also genotype × time interaction (*F* (3, 41) 4.11, *p* = 0.01). Linear regression revealed significantly different slopes by genotype (*F* (1, 67) 9.01, *p* = 0.004, *n* = 3–14/genotype), with the slope of Tie2hsEH ventricle/ brain being different from zero (*F* (1, 29) 16.59, *p* = 0.0003) while the slope of WT ventricle/brain was not, indicating that ventricle/ brain ratio increases with age in Tie2hsEH, but not WT, mice, indicative of premature brain aging.

Measuring absolute ventricular volume, there was a Tie2hsEH-specific decrease in volume across the ventricular system in the 7-month-old cohort (*F* (1, 18) 13.11, *p* = 0.002), but not in the 16-month-old cohort, there was also a CCAO-induced reduction in the 7-month-old cohort (*F* (1, 18) 4.791, *p* = 0.042), but not in the 16-month-old cohort. Pooling sham and CCAO values, ventricular system volume was reduced from 12.32 ± 0.43 mm^3^ in WT brains to 10.20 ± 0.85 mm^3^ in Tie2hsEH in the 7-month-old cohort (Fig. 4c). This reduction across the ventricular system appeared to be due to a decrease in volume of the lateral ventricle, which was also reduced in Tie2hsEH brains compared to WT in the 7-month (*F* (1, 18) 16.13, *p* = 0.001; Fig. 4e), but not in the16-month cohort (Fig. 4f); other components of the ventricular system were unaffected by either genotype or CCAO surgery (*n* = 5–7/genotype/treatment; Supplementary Fig. 3). Additionally, in the 7-month-old cohort, there was a significant decrease in volume of the lateral ventricles in after CCAO surgery across genotypes (*F* (1, 18) 6.86, *p* = 0.02). Representative images are shown in Fig. 4g demonstrating differences, particularly in the lateral ventricles, in the WT CCAO versus Tie2hsEH CCAO brain at 7 months of age. Our results are suggestive of premature aging of the Tie2hsEH brain, with reduced brain volume and increased ventricle/brain ratio in Tie2hsEH mice compared to WT, and differences in absolute ventricle volume between genotypes in the young cohort, but no longer in the aged.

During MRI of the 7-month-old cohort (4 months following surgery), ventriculomegaly was observed in 2 of the 13 Tie2hsEH mice (1 sham, 1 CCAO), both mice were excluded from further analysis. In order to determine whether this was a genotype effect, rather than an increased susceptibility from either sham or CCAO surgery, mice entering the study in the older cohort were assessed by T_2_-weighted MRI prior to surgery. In these 12-month-old mice, none of the 16 WT mice scanned exhibited hydrocephalus; however, 3 of the 18 Tie2hsEH mice had severe hydrocephalus. T_2_-weighted images from these 3 mice are shown in Fig. 4h.

### Increased leukocyte infiltration in Tie2hsEH mice

**Fig. 5:**
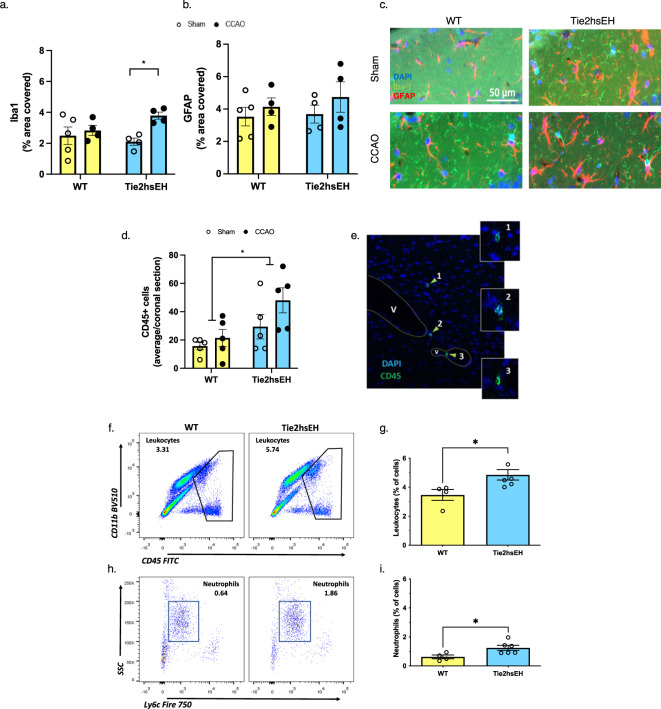
Increased leukocyte infiltration in Tie2hsEH mice. CD45, Iba1 and GFAP were assessed by immunohistochemistry, leukocytes and neutrophils by flow cytometry, in 7-month-old mice. Iba1 immunoreactivity is increased in fornix by CCAO in Tie2hsEH mice, but not in WT (**a**). GFAP levels are unaffected in fornix by either genotype or surgery (**b**); representative images of GFAP (red) and Iba1 (green) immunolabelling (**c**). CD45-positive cells within brain parenchyma are increased in Tie2hsEH vs. WT (**d**); expression is unaffected by CCAO surgery, **e** representative image of CD45-positive cells by immunolabeling. Genotype-specific increase in leukocytes is also observed in naïve mice by flow cytometry; infiltrating leukocytes were determined by high expression of CD45, utilizing CD11b staining to improve resolution of leukocytes from microglia (**f**, **g**). Further analysis reveals that this increase is characterized by selective recruitment of neutrophils, defined as CD45^hi^CD11b + CD11c-Ly6c^mid^SSC^hi^ (**h**, **i**). *p < 0.05, 2-way ANOVA with Sidak’s multiple comparisons test (histology), two-tailed *t*-test (flow cytometry), *n* = 4–6/group, data are represented as mean ± SEM.

Vascular cognitive impairments in humans are commonly associated with neuroinflammation, and white matter damage in particular^7,15,16^. Since EETs have established anti-inflammatory properties^4^, we next examined markers of neuroinflammation in the brain by immunohistochemistry (IHC) and flow cytometry. To assess glial activation, we investigated the microglia/ macrophage marker Iba1, and the astrocyte marker GFAP, in the fornix, the major white matter output tract extending from hippocampus, in the 7-month-old cohort. There were no differences between the two genotypes in level of expression of either Iba1 (*n* = 4–5/group; Fig. 5a) or GFAP (*n* = 4–5/group; Fig. 5b). Iba1 was increased by CCAO surgery (*F* (1,13) 6.19, *p* = 0.03), with Sidak’s post-hoc analysis revealing increased expression in Tie2hsEH CCAO versus Tie2hsEH sham, but not in the WT groups, suggesting that endothelial expression of sEH sensitizes white matter to hypoperfusion-induced microglial activation. GFAP expression was unaffected by surgery in either genotype. This finding suggests that neither astrocytic, nor microglial, activation account for the cognitive impairment in Tie2hsEH mice. Therefore, we next investigated infiltration of peripheral leukocytes into the brain, as a consequence of endothelial dysfunction, as a possible underlying mechanism.

The leukocyte marker CD45 was assessed throughout coronal brain sections using IHC in both the 7-month and 16-month cohorts. In both cohorts, there was a genotype-specific effect on CD45 immunoreactivity, with increased incidence of CD45-positive cells throughout brain parenchyma in Tie2hsEH compared to WT mice, with no effect of CCAO (Fig. 5d, Supplementary Fig. 4). In the 7-month mice, CD45 immunoreactivity per coronal section was increased from 18.50 ± 2.90 cells in WT to 38.70 ± 9.30 in Tie2hsEH (*F* (1,16) 8.27, *p* = 0.01, *n* = 5/genotype/treatment). To further characterize this genotype-specific increase in CD45 cells, we carried out flow cytometry on naïve 7-month-old brains from WT and Tie2hsEH mice (gating strategy shown in Supplementary Fig. 5). Leukocyte (CD45^hi^) frequency was significantly increased in Tie2hsEH compared to WT mice (3.47 ± 0.37% vs. 4.85 ± 0.36%; *p* = 0.03, *n* = 4–6 mice/genotype; Fig. 5f, g). Furthermore, the increase in leukocytes was largely due to increased neutrophil numbers (CD45^hi^CD11b^+^CD11c^-^Ly6c^mid^SSC^hi^) in Tie2hsEH brains compared to WT brains (*p* = 0.03, *n* = 4–6 mice/genotype; Fig. 5h, g). Together, these results indicate that infiltrating leukocytes, symbolic of a dysfunctional endothelium, rather than glial activation, is associated with the cognitive deficits observed in Tie2hsEH mice. To determine whether gross blood brain barrier leakage may underly the leukocyte infiltration into the brain observed in Tie2hsEH mice, we investigated the ability of 2 fluorescent markers to enter the parenchyma from the circulation (Supplementary Fig. 6). Markers of 2 molecular sizes were used to determine whether paracellular (66 kDa) or transcellular (3 kDa) leakage occur. We found that permeability to either dye was not different in Tie2hsEH mice compared to WT (66 kDa: 100.6 ± 9.15 vs. 94.68 ± 7.75; 3 kDa: 91.98 ± 1.54 vs. 83.99 ± 4.19 mean gray value. respectively, *n/s, n* = 3 mice/group).

## Discussion

Our study demonstrates that transgenic endothelial expression of human sEH induces cognitive impairment, associated with brain atrophy, leukocyte infiltration and age-dependent ventriculomegaly. This is the first clear demonstration that an endothelial mechanism is sufficient to induce three hallmark features of aging-related dementia due to vascular causes: cognitive impairment, neuroinflammation and hydrocephalus. Importantly, Tie2hsEH mice recapitulate observations made in postmortem human brain tissue with a history of dementia and small vessel disease, where we observed increased endothelial sEH expression and increased 14,15-DHET^7^. These observations were also made in mice on chronic high-fat diet that also develop cognitive impairment linked to vascular and inflammatory mechanisms^5^. As such, the Tie2-hsEH mouse represents a genetic model of aging-related VCI, and may aid in understanding its underlying pathophysiology. Our findings also identify endothelial dysfunction and sEH as potential upstream targets for the development of VCI therapeutics.

Our study indicates that endothelial dysfunction in Tie2-hsEH mice is linked to reduced endothelial EETs. This conclusion is supported by measurements of endothelial DHETs and plasma EETs. Because EETs are potent vasodilators, we first investigated hypoperfusion as a potential consequence of endothelial dysfunction and reduced endothelial EETs. However, neither global nor hippocampal CBF is affected by endothelial sEH upregulation. We next investigated neuroinflammation as an alternative manifestation of endothelial dysfunction and reduced EETs, based on reported anti-inflammatory properties of EETs^4^. We found that glial activation markers were not increased in Tie2hsEH versus WT mice, although we did observe an increase in Iba1 in Tie2hsEH mice following CCAO. Our gross analysis of both GFAP and Iba1 coverage however does not discount that there may be more subtle differences in the morphology of these two cell types; a more detailed analysis would be required to ascertain this. However, we observed an increase in infiltrating leukocytes and neutrophils. This is consistent with blood–brain barrier (BBB) dysfunction as a potential mechanism underlying cognitive impairments and subsequent leukocyte infiltration. Maintaining optimal conditions for neuronal function rests in part on an intact, quiescent endothelium^17^, and changes in endothelial permeability predispose to neuroinflammation and neurodegeneration^18,19^. BBB dysfunction leads to extravasation of plasma proteins, including hemoglobin and plasma-derived proteins such as albumin, plasmin, thrombin, fibrin, immunoglobulins and others. Plasma-derived proteins elicit a local inflammatory reaction that leads to the production of reactive oxygen species, proinflammatory cytokines and matrix metalloproteinases (MMPs), which aggravate BBB breakdown and induce expression of adhesion molecules in endothelial cells, contributing to leukocyte adhesion and extravasation^20^.

A leukocyte marker, CD45 labels cells of both myeloid and lymphoid origin; both have recently been linked to cognitive impairment^21,22^. We demonstrate that a large proportion of the increase in leukocyte numbers is attributed to an increase in myeloid cells; specifically neutrophils. Neutrophils are recruited to brain tissue by increased endothelial P-selectin expression^23^ and lead to further compromise of the BBB and local toxicity by degranulation and release of reactive oxygen species (ROS) and neutrophil extracellular traps (NETs)^24^, and may therefore contribute to the pathogenesis of the Tie2hsEH phenotype observed in this study. Indeed, neutrophil depletion in AD mice has been shown to reduce neuropathology and improve memory^25^. In elderly humans, BBB breakdown is associated with cognitive decline and inflammation, in a manner suggested to involve neutrophil migration^26^. Interestingly, circulating neutrophil phenotype and neutrophil-to-lymphocyte ratio is linked to the rate of cognitive decline in AD, with the latter potentially serving as a diagnostic biomarker^27,28^. Studies have shown that blockade of sEH reduces neuropathology-associated inflammation^29,30^, resulting in cognitive improvement in the bilateral carotid artery stenosis (BCAS) model of VCI^31^, however these studies do not inform as to whether sEH is sufficient to induce VCI, or whether it is upregulated as a consequence of VCI, nor do they inform us of the cell type responsible for the observed improvement in outcome. We have previously reported increased sEH in human brain microvascular endothelium in VCI and in brain tissue brought about by CCAO in mice^7^. We demonstrate here that sEH upregulation in the endothelium is sufficient to induce VCI, and the related neuroinflammation. Mechanistically, increased leukocytes and neutrophils in brain parenchyma as a result of sEH upregulation in the endothelium is likely linked to adhesion molecule upregulation. Upregulation of sEH is associated with an increase in vascular cell adhesion molecule (VCAM-1) and intercellular adhesion molecule 1 (ICAM-1)^32^. We have also demonstrated reduced VCAM-1 expression in sEH knockout mouse brain following subarachnoid hemorrhage (SAH)^33^. Therefore, it is possible that endothelium with increased sEH express higher levels of endothelial adhesion molecules, leading to attachment and subsequent infiltration of leukocytes, including neutrophils, into the brain, despite no evidence of gross BBB leakage.

Hippocampal volume and synaptic density were not altered by endothelial expression of sEH, but brain volume was reduced in Tie2hsEH versus WT mice, with both genotypes showing age-dependent brain atrophy, suggesting premature brain atrophy in Tie2hsEH mice. Another indication of brain atrophy observed in Tie2hsEH mice is ventriculomegaly and an increase in ventricle/ brain ratio. A similar phenomenon is observed in humans^13^. As the brain shrinks in older individuals or those with Alzheimer’s disease, CSF volume increases to fill the extra space.

Because EETs are vasodilators^4,9^, we first determined if cognitive impairment in Tie2-hsEH mice is linked to reduced CBF. However, we found no differences in global or hemispheric CBF. We did observe a decrease in ipsilateral hippocampal blood flow induced by CCAO in both genotypes, confirming mild surgery-induced hypoperfusion. However, hippocampal blood flow was not different between Tie2hsEH and WT mice, indicating that the cognitive impairment in the Tie2hsEH mice is unrelated to decreased hippocampal blood flow. The small decrease in ipsilateral hippocampal blood flow may, however, contribute to the deficits observed in the CCAO groups. The hippocampus has lower resting blood flow and oxygenation than cortex, as well as a diminished hyperemic response to neuronal activity, underlying its increased vulnerability to damage in disease states^34^. A small decrease in hippocampal blood flow, as seen here by CCAO, is likely sufficient to lead to cognitive impairment. Although resting CBF is unaltered by endothelial expression of human sEH, regulation of evoked CBF, cannot be discounted as a potential mechanism for the genotype differences observed. We have previously shown that endothelial expression of human sEH abolishes CBF responses to whisker- and acetylcholine-stimulation^8,9^; we demonstrated that the capillary response to functional hyperemia is attenuated in Tie2hsEH mice, a phenomenon likely due to decreased action of endothelial-derived EETs on capillary pericytes^9^. Also, pharmacologically induced neurovascular uncoupling, by simultaneous inhibition of CYP, nitric oxide and cyclooxygenase (COX), is associated with cognitive impairment in mice^35^. While resting CBF may be unaltered by genotype, the impaired endothelial response to stimuli and therefore neurovascular coupling due to sEH upregulation may play a role in the behavioral deficits we observe.

Although the hippocampus is more vulnerable to ischemic damage^34^, we did not observe any differences in hippocampal volume indicative of atrophy, nor did we see any changes in synaptic density between surgery groups or genotype. We have previously observed hippocampal atrophy at a later time-point following CCAO surgery, 7 months versus 4 months in this study^10^. Seven months was chosen previously as frank atrophy develops subsequent to the initial neuronal damage which manifests as cognitive impairment. Because this may develop in our CCAO groups over time, we also assessed 16-month-old mice. However, in the aged cohort, Tie2hsEH hippocampal volume was not different from that of the WT, indicating that hippocampal damage is not the likely mechanism for the cognitive deficits observed in the Tie2hsEH mice.

We demonstrate reduced endothelial EETs in Tie2hsEH mice which, together with published studies linking EETs to underlying mechanisms as discussed, leads us to postulate underly the phenotype observed. However, sEH also hydrolyzes other polyunsaturated fatty acid epoxides (EpFA), namely epoxyeicosatetranoic (EEQ) and epoxydocosapentaenoic (EDP) acids, into their less active diols^36^. Studies into these omega-3 epoxides in the brain and cerebral vasculature in particular remains sparse, although they do also demonstrate vasoactive and anti-inflammatory properties in other organ systems^37–44^, which may contribute to the phenotype observed here. Further study into omega-3 epoxide levels in Tie2hsEH mice, and their functions in brain physiology is required. Again, due to the reduced endothelial EETs we observed, and increased plasma DHETs, we postulate that the hydrolase function of sEH underlies the phenotype we describe. Soluble epoxide hydrolase, however, is a heterodimer possessing a much-studied C-terminal hydrolase domain as well as a lesser-studied N-terminal phosphatase domain^45,46^. The latter may possess vasoactive properties by mediating eNOS activity^47^, and may also underly discrepancies observed in studies of cerebral inflammation following stroke between genetic deletion of sEH and pharmacological inhibition targeting the hydrolase domain specifically^48^. It is therefore possible that increased phosphatase activity in endothelium of Tie2hsEH mice may contribute to the phenotype we describe in this study.

Males are at higher risk of developing VCI^11,12^, male mice were used in this study. Expression of sEH is sexually dimorphic in the brain, with higher levels in the male than female brain, corresponding with higher levels of 14,15 EET metabolite, 14,15-DHET in male plasma^49^. This is also true of cerebral endothelial cells in vitro, with higher levels of EPHX2, the gene encoding sEH, and lower cellular EETs in male- than female-derived cell cultures^50^. These differences in sEH levels contribute to sex differences in endothelial function, blood flow, and stroke outcome^51^. Since males already have high levels of endothelial EETs, the effect of transgenic expression of human sEH in this study may show a milder phenotype than transgenic expression in females who have lower levels. Further studies remain to be carried out to ascertain whether the cognitive impairment, ventriculomegaly and leukocyte infiltration observed in this study in males, may be exacerbated in females.

In conclusion, we demonstrate that transgenic expression of human sEH specifically in endothelium is sufficient to induce cognitive impairment, recapitulating features of the human VCI phenotype. We demonstrate that Tie2hsEH mice suffer cognitive impairment at baseline and display a more severe cognitive phenotype than WT mice after CCAO. We show that mice with increased endothelial sEH have smaller brains than WT mice, accelerated age-dependent ventriculomegaly and increased neuroinflammation attributed to infiltration of peripheral leukocytes. Our study also suggests that the cognitive impairment observed in the Tie2hsEH mice are independent of changes in resting CBF. Given that the cerebral vasculature is more accessible to drugs than other brain cell types, we propose endothelial sEH as a potential therapeutic target in VCI.

## Online methods

### Animals

This study was conducted in accordance with the National Institutes of Health guidelines for the care and use of animals in research, and protocols were approved by the Institutional Animal Care and Use Committee at Oregon Health and Science University, Portland, OR, USA. Reporting of results conforms to ARRIVE (Animal Research Reporting in In Vivo Experiments) guidelines. Adult male mice (3 months ± 2 weeks, or 1 year old ± 2 weeks) were used, as specified. Mice with endothelial-specific over-expression of human sEH (Tie2hsEH) were used, and their littermate controls^8^.

### Unilateral common carotid artery occlusion (CCAO)

CCAO or sham surgery was performed in 3- and 12-month-old male WT and Tie2hsEH littermates. Mice were anesthetized using 2% isoflurane and kept warm with water pads. Following midline cervical incision, the right common carotid artery was isolated and two 6-0 silk sutures were placed beneath the vessel. For CCAO surgeries, the sutures were tightly tied, the carotid artery was cauterized between the two sutures and cut. For sham surgeries, the sutures were removed without being tied and vessels were not cauterized. For both groups, the incisions were closed and mice were allowed to recover. Surgery survival rate was 100%.

### Behavioral testing

Behavioral testing was conducted 3 months after sham or CCAO surgery. Mice were tested over 14 days. Mice were first tested for sensorimotor function and anxiety-related using open field and rotarod test. Mice were subsequently tested for non-spatial learning in the novel object test, and spatial learning and memory in the water maze. Finally, mice were tested for contextual and cued fear conditioning. The tests were performed as described below in detail.

#### Open field

Mice were placed into a square arena. The total open field is 16 × 16 inches, the center square is 8 × 8 inches. Mice were allowed to explore for 10 min. Performance was tracked and scored using an automated video system (Ethovision 7.0 XT, Noldus Information Technology, Wageningen, Netherlands). Time spent in the center of the open field was analyzed.

##### Novel object recognition

Mice were habituated to the open field above over 3 days with one 10-min trial per day. On day 4, the mice were exposed to the arena containing two identical objects. On day 5, one of the “familiar” objects was replaced by a novel object. Performance of the mice was video recorded. Orientation to the object, within 2 cm proximity, as well as interaction with the object (climbing, sniffing, pushing) was defined as exploring the object. Novel object recognition and discrimination was calculated as the percent time spent exploring the novel object out of the total time spent exploring both objects.

##### Rotarod

Sensorimotor performance was assessed on a rotarod. Mice were placed on an elevated rotating rod (diameter: 3 cm, elevated: 45 cm, Rotamex-5, Columbus Instruments, Columbus, OH, USA), initially rotating at 5.0 rpm. The rod accelerated 1.0 rpm every 3 s. A line of photobeams beneath the rod recorded the latency to fall (seconds). Each mouse received three trials per day, with no delay between trials, on 3 consecutive days.

##### Water maze

Hippocampus-dependent spatial learning and memory was assessed in the water maze. The maze consisted of a circular pool (diameter 140 cm), filled with opaque water (24 °C), divided conceptually into four quadrants. Mice were first trained to locate an “escape” platform (plexiglass circle, 6 cm radius) submerged 2 cm below the surface of the water and made visible by the use of a cue (a colored cylinder, 2.5 cm radius, 8 cm height) during the “visible” trials (days 1 and 2). For the visible platform training days, there were two daily sessions, morning and afternoon, which were separated by an intersession interval of 2 h. Each session consisted of three trials, with 10-min inter-trial intervals. Mice were placed into the water facing the edge of the pool in one of nine randomized locations (consistent for each mouse). A trial ended when the mouse located the platform. Mice that failed to locate the platform within 60 s were led to the platform by placing a finger in front of their swim path. Mice were taken out of the pool after they remained on the platform for a minimum of 10 s.

During the visible platform sessions, the location of the platform was moved between each of the four quadrants to avoid procedural biases in task learning. Subsequent to the visual trials, mice were trained to locate a hidden platform, requiring the mice to rely on extra maze cues for spatial reference and orientation. The platform was not rotated during the hidden platform trials and remained in the same location. Twenty-four and seventy-two hours after the last hidden platform training trial, spatial memory retention of the mice was assessed in a “probe” trial (no platform). During the probe trials, mice were placed into the water in the quadrant opposite of the target quadrant. The time spent in the target quadrant compared to the time spent in the three non-target quadrants was analyzed.

The swimming patterns of the mice were recorded with Noldus Ethovision video tracking software (Ethovision 7.0 XT, Noldus Information Technology, Wageningen, Netherlands) set at six samples/s. The time to locate the platform (latency) was used as a measure of performance for the visible and hidden platform sessions. Latency to reach the target was measured in seconds, and was calculated for each day by averaging values from the six daily trials. Because swim speeds can influence the time it takes to reach the platform, they were also analyzed.

##### Fear conditioning

In this task, mice learn to associate a conditioned stimulus (CS, e.g. the environmental context, or a discrete cue) with a mild foot shock (unconditioned stimulus, US). CS–US pairings are preceded by a short habituation period, during which a baseline measure of locomotor activity is analyzed. Contextual fear conditioning is considered to be hippocampus- and amygdala-dependent, while fear conditioning is considered to be hippocampus independent. Freezing, defined as immobility with the exception of respiration, is considered a post-exposure fear response, and is a widely used indicator of conditioned fear.

Mice were trained and tested using a Med Associates mouse fear conditioning system and VideoFreeze automated scoring system (Med Associates, St. Albans, Vermont),^52^. On day 1, the mice were placed inside a dark fear-conditioning chamber. Chamber lights (at 100 lux) turned on at zero seconds, followed by a 90-s habituation period and a subsequent 30-s (2800 Hz, 80 dB) tone (cue). A 2-s 0.7 mA footshock was administered at 28 s, co-terminating with the tone at 30 s. After a 30-s inter-stimulus-interval the tone-shock pairing were repeated for a total of five tone-shock pairings. On day 2, hippocampus dependent associative learning was assessed during re-exposure to the training environment for 300 s. 3 h later, mice were exposed to a modified environment (scented with vanilla extract, cleaned with 10% isopropanol instead of 0.5% glacial acetic acid, novel floor texture covering the shock-grid, and rounded walls). They were allowed to habituate for 90 s, and then exposed to the cue for a second period of 180 s. Associative learning was measured as the percent time spent freezing in response to the contextual environment or the tone. Immediate acquisition of conditioned fear was measured following CS–US pairings. Motion during shock (proprietary index, Med Associates) was measured to account for potential differences in response to the shock during training.

### Magnetic resonance imaging (MRI)

Imaging was conducted after completion of behavioral testing, 4 months following sham or CCAO surgery.

#### MRI data acquisition

MR imaging was performed at the OHSU Advanced Imaging Research Center using a Bruker-Biospin 11.75 T small animal MR system with a Paravision 5.1 software platform, 10-cm inner diameter gradient set. For arterial spin labeling (ASL) MRI and associated T_2_ weighted imaging, a 72 mm (ID) RF resonator for transmit and an actively decoupled mouse head surface coil for receive. For studies employing only T_2_ weighted imaging, MR imaging employed a mouse head (20 mm ID) quadrature RF transceiver coil (M2M Imaging Corp.) Mice were anesthetized with a ketamine/xylazine mixture (1.0 mg xylazine/7 mg ketamine/100 g) in combination with low isoflurane (0.75%) in 100% oxygen. The mice were positioned with heads immobilized on an animal cradle. Body temperature of the mice was monitored and maintained at 37 °C while monitoring respiration. For the ASL MRI studies, a coronal 25-slice T_2_-weighted image was obtained (Paravision spin echo RARE, 256 × 256 matrix, 125 µm in-plane resolution, 0.5 mm slice width, TR 4000 ms, TE effective 46.82 ms, RARE factor 8, 2 averages). These T_2_-weighted anatomical scans were used for positioning the perfusion image slice at a consistent position approximately 1.5 mm posterior to the anterior commissure. CBF (ml/min/100 g) was measured using arterial spin labeling (ASL), employing the flow-sensitive alternating inversion recovery rapid acquisition with relaxation enhancement pulse sequence (Paravision FAIR-RARE), with TE/TR = 45.2/10,000 ms, slice thickness = 2 mm, number of slices = 1, matrix = 128 × 128, 250 µm in-plane resolution RARE factor = 72, and 23 turbo inversion recovery values ranging from 40 to 4400 ms, acquisition time 15 min. This sequence labels the inflowing blood by global inversion of the equilibrium magnetization^53^. Studies employing only T_2_-weighted imaging employed the parameters above with the exception of the in-plane resolution (112 µm) and TEeffective (23.6 ms).

#### MRI data analysis

Images were analyzed using the Bruker Paravision software and JIM software (Xinapse Systems LTD, Northants UK). CBF maps (ml/100g-min) were generated using the Bruker ASL perfusion processing macro and exported into JIM for further processing. Outlier value brain pixels (outside 2 SDs) representing large arteries with high, pulsatile flow were excluded, thus arriving at flows which consistently represent tissue microvascular flow. The identical FOV geometry offsets of the T_2_ and ASL images enabled the ROIs drawn on the T_2_ to be readily overlaid onto the corresponding perfusion map for quantification. Mean CBF was quantified for the whole brain and each hemisphere each defined anatomically using the corresponding T_2_ images. Ventricular regions were excluded from analysis if present within the ASL slice. Hippocampal CBF was determined as follows: hippocampal regions were outlined on the T_2_ image slices that were within the ASL image slice. The intersecting areas of these outlines were used to determine regions within the ASL image slice that are completely hippocampal. The mean CBF within this intersecting hippocampal region was determined. The mean hippocampal CBF was corrected for the volume fraction of ventricle within the intersecting region, if any (ventricular components were assumed to have zero CBF). Brain size was quantified from brain outlines drawn on the T_2_ image slices, between 3 mm anterior, and 7 mm posterior, to the anterior commissure.

### Histology

Mice were sacrificed 4 months following surgery; they were perfused with heparinized cold saline followed by 4% cold paraformaldehyde (PFA). After removal from the skull, brains were post-fixed overnight in 4% PFA, dehydrated and cleared (Prosoft and Propar, Anatech Ltd, Battle Creek, MI) for paraffin embedding. Serial 6 μm-thick sections were cut and deparaffinized sections underwent antigen retrieval (heated in 10 mM citrate buffer at pH 6.0 in a steamer for 30 min) and subsequently blocked with 4% normal donkey serum in phosphate-buffered saline with 1% bovine serum albumin and 0.3% Triton for 60 min at room temperature to prevent nonspecific binding of the secondary antibodies. For GFAP, sections were blocked using the Vector Mouse-on-Mouse kit (BMK-2202, Vector Laboratories, Burlingame, CA). Sections were incubated with primary antibodies (mouse anti-glial fibrillary acidic protein (GFAP), clone GA5, 1:500, #MAB360 EMD Millipore, Billerica, MA; rabbit polyclonal anti-ionized calcium binding adaptor molecule 1 (Iba-1) 1:1000, #019-19741 Wako Chemicals, Richmond, VA; rat anti-mouse CD45 1:500, #550539 BD Biosciences, rabbit monoclonal anti-synaptophysin 1:500, #ab32127, abcam) diluted in the blocking serum, overnight at 4 °C. The fluorescent-labeled secondary antibodies (Alexa Fluor 594-conjugated donkey anti-mouse 1:250, # A2120; Alexa Fluor conjugated donkey anti-rabbit 1:500, #A21206 (both Life Technologies, Eugene, OR); rhodamine conjugated donkey anti-rabbit 1:300, #711-295-152 (Jackson ImmunoResearch, West Grove, PA) diluted in the blocking serum, were applied to the sections for 150 min at room temperature. The sections were counterstained with Hoechst 33342 (Molecular Probes, 1:3000, Eugene, OR), and coverslipped with Fluoromount-G (Southern Biotech, Birmingham, AL). Fluorescent images were acquired on a Zeiss LSM 710 confocal microscope. All analysis was conducted by experimenters blinded to both surgical group and genotype using Image J software. For quantification of CD45 cells were counted manually over the entirety of the coronal section; 3 serial sections were quantified and averaged per brain. For quantification density of Iba-1, GFAP and synaptophysin, a threshold was set in Image J area covered within an ROI of a standard size was calculated for each specified brain region.

### Flow cytometry

Prior to tissue harvest, animals were anesthetized with isofluorane and intracardially perfused with cold heparinized saline. After removing the cerebellum and olfactory bulbs, brain tissue was processed immediately by fine cutting followed by digestion with an enzyme cocktail of collagenase/DNase (Sigma). Myelin was removed by density centrifugation in DMEM supplemented with 20% BSA, and the remaining cell pellet was resuspended in media for staining. Brains were processed individually, without pooling. Isolated cells were first blocked using CD16/CD32 Mouse BD Fc Block (BD Biosciences, San Jose, CA), and surface proteins were detected using monoclonal antibodies CD45 FITC (30-F11, Biolegend, San Diego, CA), CD11b BV510 (M1/70, Biolegend), CCR2 BV650 (SA203G11, Biolegend), CD3e PE (145-2C11, BD Pharmingen), CD19 PE-Cy7 (6D5, Biolegend), CD11c APC (N418, Biolegend), and Ly6c APC-Fire750 (HK1.4, Biolegend), CD74 BV421 (In1, BD). Dead cells were excluded using Live/Dead Fixable Blue (Thermo Fisher Scientific, Waltham, MA), according to manufacturer’s instructions. After staining, cells were washed and acquired using a FACSymphony (BD Scientific) equipped with FACS Diva v.8, and data were analyzed using FlowJo software v.10.0.7.

### Assessment of blood–brain barrier permeability

FITC-albumin (66 kDa; Sigma Aldrich) and rhodamine-dextran (3 kDa, ThermoFisher Scientific), 2 mg/0.1 ml each, were intravenously administered through the jugular vein and allowed to circulate for 60 min prior to sacrifice. As a positive control, the same procedure was carried out on a WT mouse 24 h after 1-h MCAO. Brains were collected without saline perfusion and postfixed in 4% PFA for 3 days. Then, brains were transferred into 10% glycerol solution for 18–24 h, 20% glycerol solution for 18–24 h, both at 4 °C. Brains were subsequently flash frozen by immersion in 2-methylbutane and sliced in a cryostat at 20 µm thick. Brain sections were collected at 4 positions: Bregma 0.98, −0.46, −2.3, and −3.52 and mounted on slides. Brain sections were then washed with phosphate-buffer saline (PB), pH 7.4 counterstained with Hoechst 33342 (trihydrochloride trihydrate, 1:50,000, Fisher Scientific Cat# H3570, USA) and mounted with Fluoromount-G (SouthernBiotech, Cat# 0100-01, Birmingham, AL, USA). Images were captured at ×20 magnification using a whole-slice laser scanning confocal microscope (Zeiss Axio Scan.Z1, Zen software). The gain and laser power settings were identical for all images acquired. Image J was used for measurement of mean gray value. Gray value is 0–255. Mean gray value is the sum of the gray values of all the pixels in the selection divided by the number of pixels. The background and vessel were segmented using the Threshold icon, and mean gray value of background and vessel were measured. Value was also normalized by lowest gray value of background. Three sections per brain were analyzed.

### Eicosanoid analysis

#### Cell DHETs

Brain microvascular endothelial cells were isolated by immune sorting with CD102 (ICAM-2) antibody-conjugated Dynabeads. Briefly, brains (devoid of pial vessels) were digested using collagenase type 2 (Worthington Biochemical, Lakewood, NJ) and triturated. Cells were pelleted, resuspended in PBS containing 0.1% BSA and incubated with CD102 antibody (clone 3C4, BD Pharmingen)-coated sheep anti-rat IgG Dynabeads (Invitrogen Life Technologies) for 40 min at room temperature. The cell-Dynabead suspension was mounted on a magnetic separator; the Dynabead-bound cells were collected and re-suspended in PBS containing anti-oxidant (butylated hydroxytoluene, 0.02 mg/mL; triphenyl phosphine, 0.2 mg/mL and indomethacin, 0.2 mg/mL), and non-bound cells discarded. Total DHET levels (free and esterified) were assessed; 15% KOH (Fisher Scientific) was added to samples, which were base hydrolyzed at 40 °C for 1 h and then acidified with 300 μl glacial acetic acid (T.J. Baker, Phillipsburg, NJ). Internal standard mix was added, and samples were extracted with ethyl acetate, hexane and hexane–ethyl acetate (1:1). Extracts were combined and dried under vacuum at 35 °C for 35 min. Tubes were washed with hexane, and samples stored at −80 °C until LC–MS/MS analysis or immediately redried, solubilized in acetonitrile and water, and analyzed. DHET standard curves were prepared in PBS and extracted identically to samples as well as an unextracted standard curve run at the same time. Area ratios were plotted, and unknowns were determined using the slopes. The slopes of the unextracted and extracted curves were very similar, and unextracted standard curves were prepared and compared with a spiked quality control media extract. Samples were analyzed by liquid chromatography–tandem mass spectrometry (LC–MS/MS) using a 5500 Q-TRAP hybrid/triple quadrupole linear ion mass spectrometer (Applied Biosystems, Carlsbad CA) with electrospray ionization in the positive mode. The mass spectrometer was interfaced to a Shimadzu (Columbia, MD) SIL-20AC XR autosampler followed by two LC-20AD XR LC pumps. Scheduled multiple reaction monitoring transitions were monitored with a 2-min window, as shown in Table 1. The gradient mobile phase was delivered at a flow rate of 0.5 ml/min and consisted of two solvents: 0.05% acetic acid in water and acetonitrile. The Betabasic-18 2 × 100-mm, 3-μm column was kept at 40 °C using the Shimadzu column oven. Data were acquired and analyzed using Analyst 1.5.1 and Multiquant 3.0.1 (AB Sciex). Eicosanoid concentration for each sample was normalized to protein concentration, determined by Pierce BCA Protein Assay Kit (Thermo Fisher Scientific); eicosanoid concentration is expressed as pg eicosanoid/ μg protein.

**Table 1:** Multiple reaction monitoring parameters for analysis of DHETs.

Compound	Retention time (min)	Q1 mass	Q3 mass	DP	EP	CE	CXP
14,15-DHET	3.60	337	207	−100	−10	−26	−3
11,12-DHET	4.00	337	167	−90	−10	−28	−11
8,9-DHET	4.40	337	127	−75	−10	−30	−9

#### Plasma EETs

Plasma samples were obtained from mice by using EDTA, with the addition of antioxidant solution (0.2 mg/mL BHT, 2 mg/mL TPP and 2 mg/mL indomethacin in methanol:ethanol 1:1; 1:100) and frozen at −80 °C until analysis. To prepare samples a 60 mg Oasis HLB cartridge was pre-equilibrated methanol with 0.2 mg/mL TPP, followed by 0.025% phosphoric acid-10% methanol. Plasma was centrifuged and diluted with 0.025% phosphoric acid and antioxidant was added. Subsequently, 1 ng of internal standard mix (1 ng d8-14,15 EET, or d11-14,15 EET, depending on commercial availability) was added. Samples were loaded onto the cartridges by gravity, with 3–5 psi positive pressure only applied in the case of a clog. Cartridges were washed with 0.025% phosphoric acid-10% methanol solution. Cartridges were dried with 3–5 psi nitrogen gas on a positive pressure unit for 15 min; the cartridge was then eluted sequentially with ethyl acetate, hexane:ethyl acetate and hexane. Trap solution (2% DMSO in methanol) was added to the combined eluates, which were then dried under vacuum for 35 min at 35 °C. The tube walls were washed with hexane and the sample was immediately re-dried until just dry (approximately 7 mins) and solubilized in 45:55 (v/v) acetonitrile:water, placed in sample vials with inserts and analyzed my LC–MS/MS. The injection volume was 30 µL. An un-extracted standard curve was run, area ratios were plotted and unknowns were determined using the slopes. A spiked and naïve control plasma prepared from commercial plasma were run routinely to confirm response and calibration. Samples were analyzed using a 5500 Q-TRAP hybrid/triple quadrupole linear ion trap mass spectrometer (Applied Biosystems) with electrospray ionization (ESI) in negative mode. The mass spectrometer was interfaced to a Shimadzu (Columbia, MD) SIL-20AC XR auto-sampler followed by 2 LC-20AD XR LC pumps and analysis on an Applied Biosystems/SCIEX Q5500 instrument (Foster City, CA). The instrument was operated with the following settings: source voltage −4000 kV, GS1 40, GS2 40, CUR 35, TEM 450 and CAD gas HIGH. Lipid standards and internal standards and were purchased from Cayman Chemical company (Ann Arbor, Michigan), Biotage Pressure + 48 Positive pressure manifold, Biotage (Uppsala, Sweden), EDTA mouse plasma were from Innovative Research (Novi, MI). Data were acquired using Analyst 1.5.1 software and analyzed using Multiquant 3.01 software. The standard curves were from 0 to 1000 pg/sample and the limit of quantification was 10 pg per sample.

### Statistics

Data are reported as mean ± SEM. Results were considered significant at *p* < 0.05. Data were analyzed using SPSS (Chicago, IL) and GraphPad Prism 9.0.1 (San Diego, CA) software. Water maze learning curves were analyzed using repeated measures ANOVA. Probe trial analyses utilized one-way ANOVAs with a Dunnet’s post-hoc test to compare non-target quadrants against the target quadrant. Rotarod performance and fear conditioning were also analyzed as repeated measures ANOVAs.

## Supplementary information

Supplementary Figures

## Data Availability

The data generated during and/or analyzed during the current study are available from the corresponding author on reasonable request

## References

[1] Iadecola C (2016). Vascular and metabolic factors in Alzheimer’s disease and related dementias: introduction. Cell. Mol. Neurobiol..

[2] Snowdon DA (1997). Brain infarction and the clinical expression of Alzheimer disease. The Nun Study. JAMA.

[3] Wang F (2018). Dysfunction of cerebrovascular endothelial cells: prelude to vascular dementia. Front. Aging Neurosci..

[4] Davis CM, Liu X, Alkayed NJ (2017). Cytochrome P450 eicosanoids in cerebrovascular function and disease. Pharmacol. Ther..

[5] Zuloaga KL (2016). High fat diet-induced diabetes in mice exacerbates cognitive deficit due to chronic hypoperfusion. J. Cereb. Blood Flow Metab..

[6] Zuloaga KL (2014). Mechanism of protection by soluble epoxide hydrolase inhibition in type 2 diabetic stroke. PLoS ONE.

[7] Nelson JW (2014). Role of soluble epoxide hydrolase in age-related vascular cognitive decline. Prostaglandins Other Lipid Mediat..

[8] Zhang W (2013). Role of endothelial soluble epoxide hydrolase in cerebrovascular function and ischemic injury. PLoS ONE.

[9] Zhang, W. et al. Role of endothelium-pericyte signaling in capillary blood flow response to neuronal activity. *J. Cereb. Blood Flow Metab*. 10.1177/0271678x211007957 (2021). 10.1177/0271678X211007957 PMC8327110 33853406

[10] Zuloaga KL (2015). Neurobehavioral and imaging correlates of hippocampal atrophy in a mouse model of vascular cognitive impairment. Transl. Stroke Res..

[11] Podcasy JL, Epperson CN (2016). Considering sex and gender in Alzheimer disease and other dementias. Dialogues Clin. Neurosci..

[12] Kim MY, Kim K, Hong CH, Lee SY, Jung YS (2018). Sex differences in cardiovascular risk factors for dementia. Biomol. Ther..

[13] Scahill RI (2003). A longitudinal study of brain volume changes in normal aging using serial registered magnetic resonance imaging. Arch. Neurol..

[14] Meyer JS, Rauch GM, Rauch RA, Haque A, Crawford K (2000). Cardiovascular and other risk factors for Alzheimer’s disease and vascular dementia. Ann. N. Y. Acad. Sci. USA.

[15] Iadecola C (2013). The pathobiology of vascular dementia. Neuron.

[16] López-Valdés HE, Martínez-Coria H (2016). The role of neuroinflammation in age-related dementias. Rev. Investig. Clin..

[17] Wyss-Coray T (2016). Ageing, neurodegeneration and brain rejuvenation. Nature.

[18] Ransohoff RM (2016). How neuroinflammation contributes to neurodegeneration. Science.

[19] Zlokovic BV (2011). Neurovascular pathways to neurodegeneration in Alzheimer’s disease and other disorders. Nat. Rev. Neurosci..

[20] Winkler EA, Sagare AP, Zlokovic BV (2014). The pericyte: a forgotten cell type with important implications for Alzheimer’s disease?. Brain Pathol..

[21] Minhas PS (2021). Restoring metabolism of myeloid cells reverses cognitive decline in ageing. Nature.

[22] Zhang Y (2020). Depletion of NK cells improves cognitive function in the Alzheimer disease mouse model. J. Immunol..

[23] Bernardes-Silva M, Anthony DC, Issekutz AC, Perry VH (2001). Recruitment of neutrophils across the blood-brain barrier: the role of E- and P-selectins. J. Cereb. Blood Flow Metab..

[24] Mayadas TN, Cullere X, Lowell CA (2014). The multifaceted functions of neutrophils. Annu. Rev. Pathol..

[25] Zenaro E (2015). Neutrophils promote Alzheimer’s disease-like pathology and cognitive decline via LFA-1 integrin. Nat. Med..

[26] Bowman GL (2018). Blood–brain barrier breakdown, neuroinflammation, and cognitive decline in older adults. Alzheimers Dement..

[27] Dong Y (2018). Neutrophil hyperactivation correlates with Alzheimer’s disease progression. Ann. Neurol..

[28] Sayed A (2020). The neutrophil-to-lymphocyte ratio in Alzheimer’s disease: Current understanding and potential applications. J Neuroimmunol.

[29] Hung TH (2017). Deletion or inhibition of soluble epoxide hydrolase protects against brain damage and reduces microglia-mediated neuroinflammation in traumatic brain injury. Oncotarget.

[30] Wu CH (2017). Genetic deletion or pharmacological inhibition of soluble epoxide hydrolase reduces brain damage and attenuates neuroinflammation after intracerebral hemorrhage. J Neuroinflammation.

[31] Chen Y (2017). Soluble epoxide hydrolase inhibition promotes white matter integrity and long-term functional recovery after chronic hypoperfusion in mice. Sci. Rep..

[32] Zhang D (2012). Homocysteine upregulates soluble epoxide hydrolase in vascular endothelium in vitro and in vivo. Circ. Res..

[33] Siler DA (2015). Soluble epoxide hydrolase in hydrocephalus, cerebral edema, and vascular inflammation after subarachnoid hemorrhage. Stroke.

[34] Shaw K (2021). Neurovascular coupling and oxygenation are decreased in hippocampus compared to neocortex because of microvascular differences. Nat. Commun..

[35] Tarantini S (2015). Pharmacologically-induced neurovascular uncoupling is associated with cognitive impairment in mice. J. Cereb. Blood Flow Metab..

[36] Morisseau C (2010). Naturally occurring monoepoxides of eicosapentaenoic acid and docosahexaenoic acid are bioactive antihyperalgesic lipids. J. Lipid Res..

[37] Ye D (2002). Cytochrome p-450 epoxygenase metabolites of docosahexaenoate potently dilate coronary arterioles by activating large-conductance calcium-activated potassium channels. J. Pharmacol. Exp. Ther..

[38] Yuan M (2021). Omega-3 polyunsaturated fatty acid supplementation improves lipid metabolism and endothelial function by providing a beneficial eicosanoid-pattern in patients with acute myocardial infarction: a randomized, controlled trial. Clin. Nutr..

[39] Ulu A (2014). An omega-3 epoxide of docosahexaenoic acid lowers blood pressure in angiotensin-II-dependent hypertension. J. Cardiovasc. Pharmacol..

[40] Yang, Y. et al. Differential effects of 17,18-EEQ and 19,20-EDP combined with soluble epoxide hydrolase inhibitor t-TUCB on diet-induced obesity in mice. *Int. J. Mol. Sci*. **22**, 10.3390/ijms22158267 (2021). 10.3390/ijms22158267 PMC8347952 34361032

[41] Schunck WH, Konkel A, Fischer R, Weylandt KH (2018). Therapeutic potential of omega-3 fatty acid-derived epoxyeicosanoids in cardiovascular and inflammatory diseases. Pharmacol. Ther..

[42] Wagner K, Vito S, Inceoglu B, Hammock BD (2014). The role of long chain fatty acids and their epoxide metabolites in nociceptive signaling. Prostaglandins Other Lipid Mediat..

[43] Wagner KM, McReynolds CB, Schmidt WK, Hammock BD (2017). Soluble epoxide hydrolase as a therapeutic target for pain, inflammatory and neurodegenerative diseases. Pharmacol. Ther..

[44] López-Vicario C (2015). Inhibition of soluble epoxide hydrolase modulates inflammation and autophagy in obese adipose tissue and liver: role for omega-3 epoxides. Proc. Natl Acad. Sci. USA.

[45] Newman JW, Morisseau C, Harris TR, Hammock BD (2003). The soluble epoxide hydrolase encoded by EPXH2 is a bifunctional enzyme with novel lipid phosphate phosphatase activity. Proc. Natl Acad. Sci. USA.

[46] Domingues MF, Callai-Silva N, Piovesan AR, Carlini CR (2019). Soluble epoxide hydrolase and brain cholesterol metabolism. Front. Mol. Neurosci..

[47] Hou HH (2012). N-terminal domain of soluble epoxide hydrolase negatively regulates the VEGF-mediated activation of endothelial nitric oxide synthase. Cardiovasc. Res..

[48] Koerner IP (2008). Soluble epoxide hydrolase: regulation by estrogen and role in the inflammatory response to cerebral ischemia. Front. Biosci..

[49] Zhang W (2009). Role of soluble epoxide hydrolase in the sex-specific vascular response to cerebral ischemia. J. Cereb. Blood Flow Metab..

[50] Gupta NC, Davis CM, Nelson JW, Young JM, Alkayed NJ (2012). Soluble epoxide hydrolase: sex differences and role in endothelial cell survival. Arterioscler. Thromb. Vasc. Biol..

[51] Davis CM, Fairbanks SL, Alkayed NJ (2013). Mechanism of the sex difference in endothelial dysfunction after stroke. Transl. Stroke Res..

[52] Anagnostaras, S. G. et al. Automated assessment of pavlovian conditioned freezing and shock reactivity in mice using the video freeze system. *Front. Behav. Neurosci*. **4**, 10.3389/fnbeh.2010.00158 (2010). 10.3389/fnbeh.2010.00158 PMC2955491 20953248

[53] Kim SG (1995). Quantification of relative cerebral blood flow change by flow-sensitive alternating inversion recovery (FAIR) technique: application to functional mapping. Magn. Reson. Med..

