# Supplementary material for: Age-dependent cognitive impairment, hydrocephalus and leukocyte infiltration in transgenic mice with endothelial expression of human EPHX2
Source: NPJ Aging. 2022 Jul 5;8(1):9. doi: 10.1038/s41514-022-00090-1 (PMC9256583; doi:10.1038/s41514-022-00090-1)
Supplement: Supplementary file 1 — Supplementary Figures [file 41514_2022_90_MOESM1_ESM.pdf]

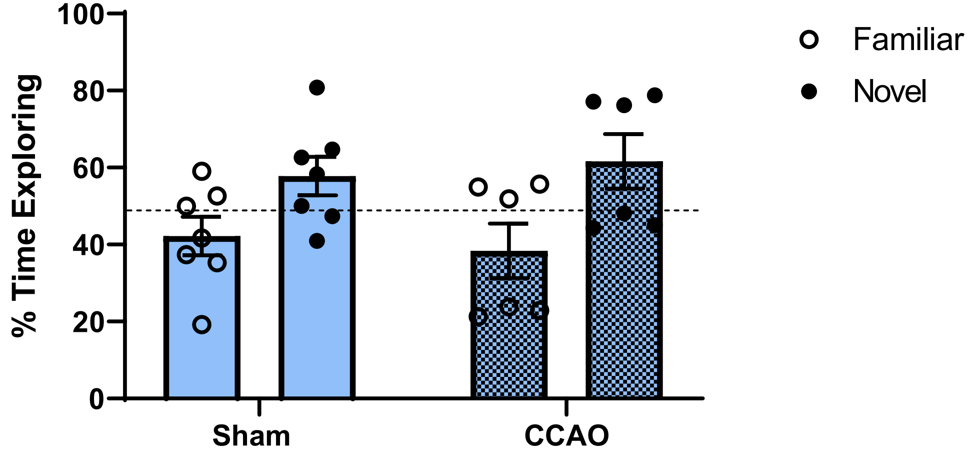

Supplementary Figure 1. Novel object recognition is unaffected by surgery in Tie2hsEH mice. Novel object recognition is intact in Tie2hsEH mice; mice spend more time exploring the novel, rather than familiar, object ($p<0.05$). Time spent exploring the novel object is not affected by CCAO surgery in Tie2hsEH mice. 2-way ANOVA, n=6-7/ group, data are represented as mean ± SEM.

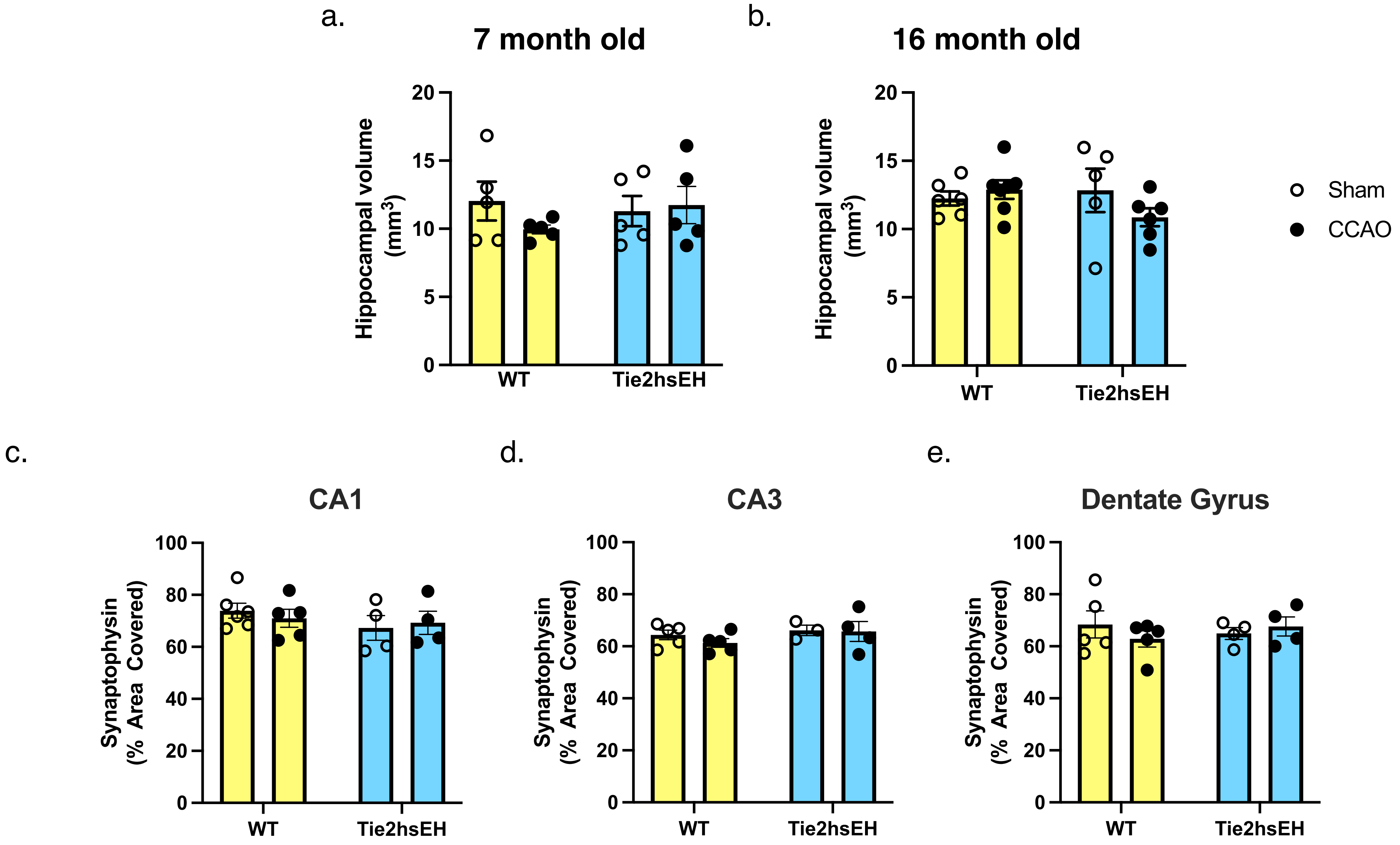

Supplementary Figure 2. Hippocampal size or synapse density are unaltered by genotype or surgery. Hippocampal size was measured by T_2-weighted MRI. Hippocampal volume is unaltered by genotype or CCAO surgery in (**a**) 7-month-old or (**b**) 16-month-old mice, n=5/group. Synapse density was assessed by immunohistochemistry for synaptophysin in 7-month-old mice. Hippocampal area covered by synaptophysin was unaffected by either genotype or surgery in (**c**) CA1, (**d**) CA3 or (**e**) dentate gyrus, n/s, 2-way ANOVA, n=3-6/ group, data are represented as mean ± SEM.

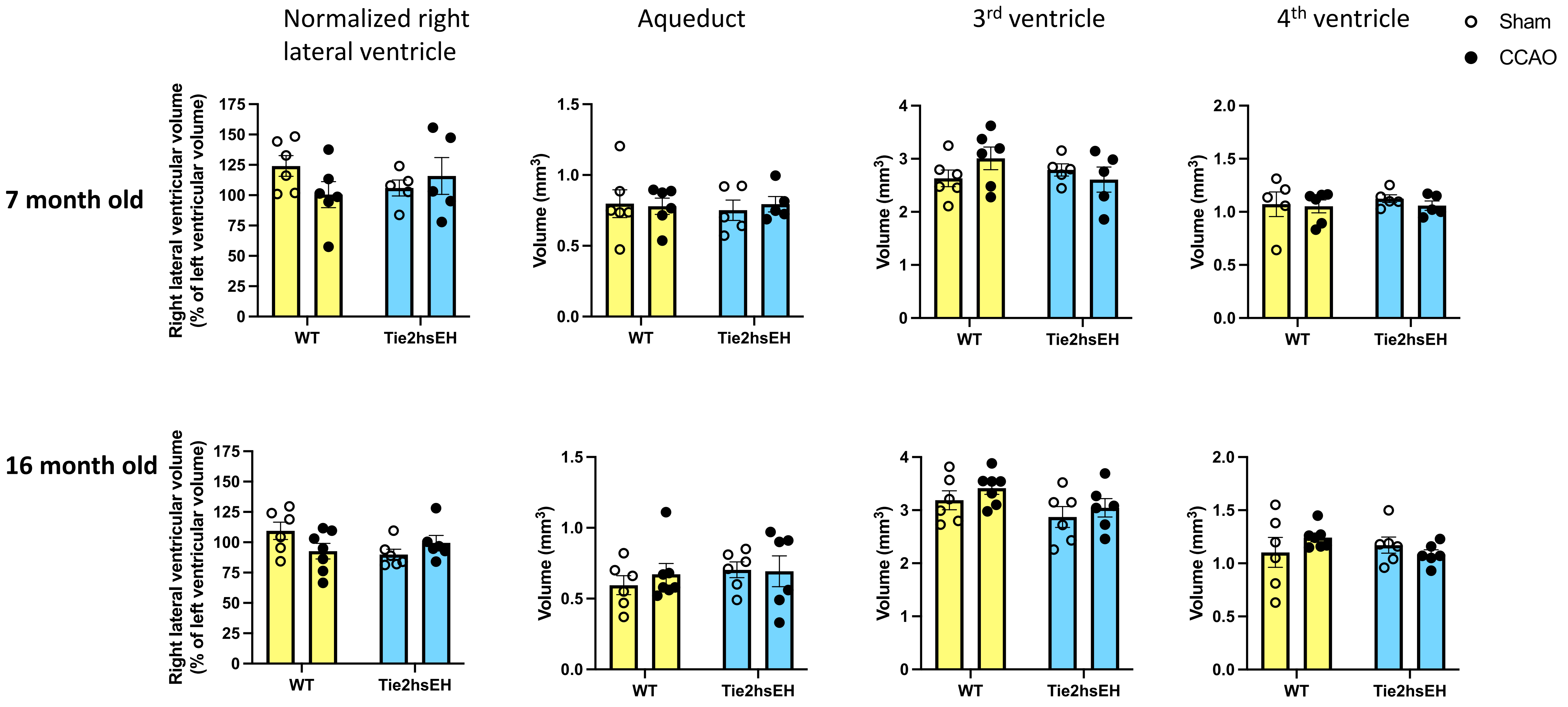

Supplementary Figure 3. MRI analysis of ventricular structures in 7- and 16-month-old mice. No differences were observed in response to CCAO surgery, as determined by normalized right ventricle volume in either age cohort. No differences were observed in aqueduct, 3rd or 4th ventricles due to either genotype or surgery, 2-way ANOVA, n=5-7/group, data are represented as mean ± SEM.

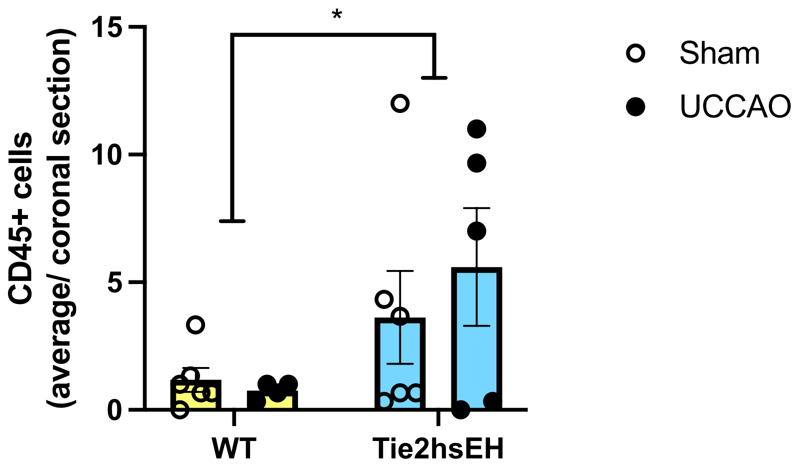

Supplementary Figure 4. Increased CD45 in 16-month-old Tie2hsEH mice. CD45 was assessed by immunohistochemistry. CD45-positive cells within brain parenchyma are increased in Tie2hsEH vs. WT 16-month-old mice; expression is unaffected by CCAO surgery. *p<0.05, 2-way ANOVA with Sidak's multiple comparisons test, n=4-6/group, data are represented as mean ± SEM.

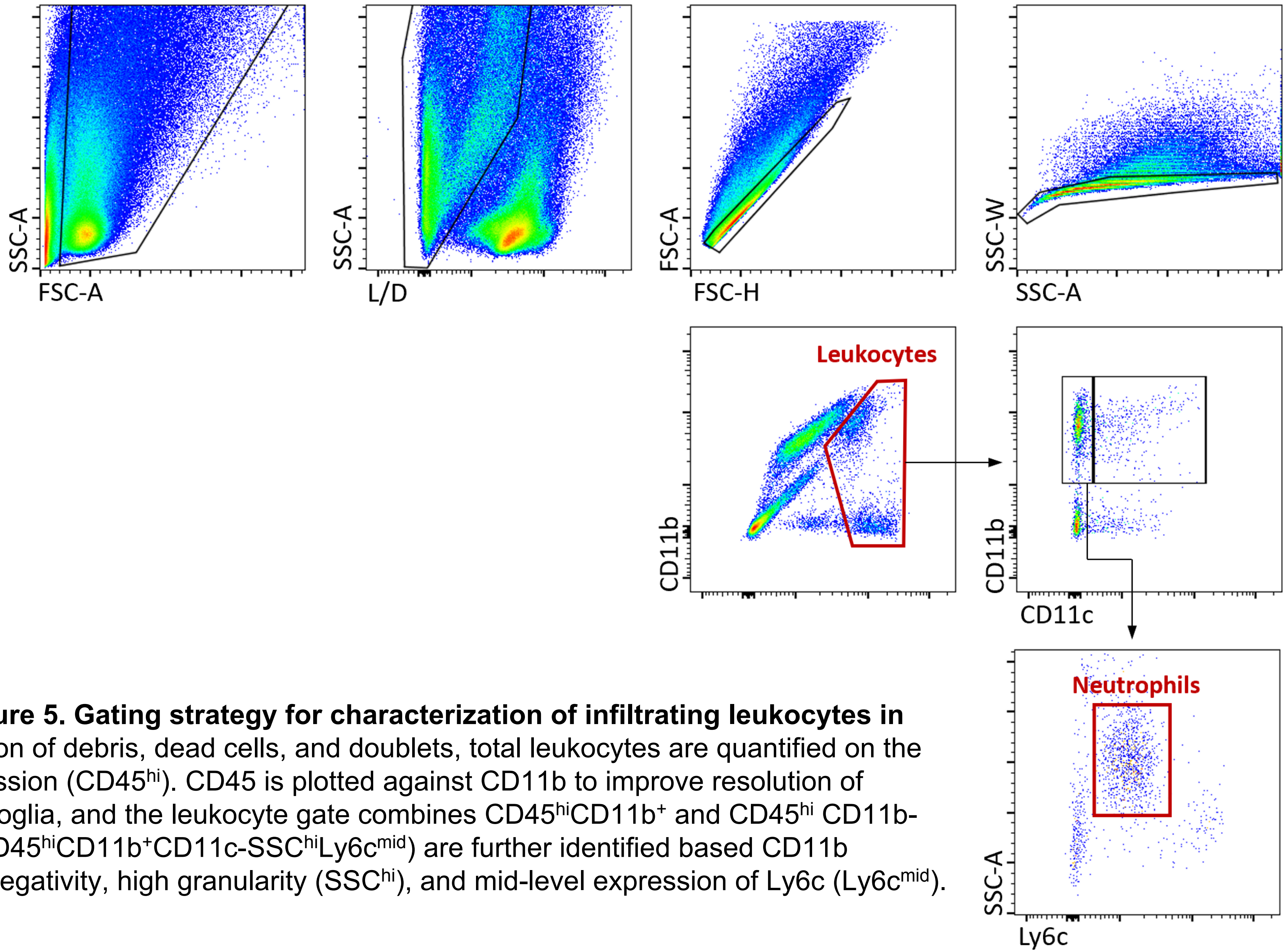

Supplementary Figure 5. Gating strategy for characterization of infiltrating leukocytes in brain. After elimination of debris, dead cells, and doublets, total leukocytes are quantified on the basis of CD45 expression ($CD45^{hi}$). CD45 is plotted against CD11b to improve resolution of leukocytes from microglia, and the leukocyte gate combines $CD45^{hi}CD11b^{+}$ and $CD45^{hi}$ CD11b- cells. Neutrophils ($CD45^{hi}CD11b^{+}CD11c\text{-}SSC^{hi}Ly6c^{mid}$) are further identified based CD11b expression, CD11c negativity, high granularity (SSC^{hi}), and mid-level expression of Ly6c ($Ly6c^{mid}$).

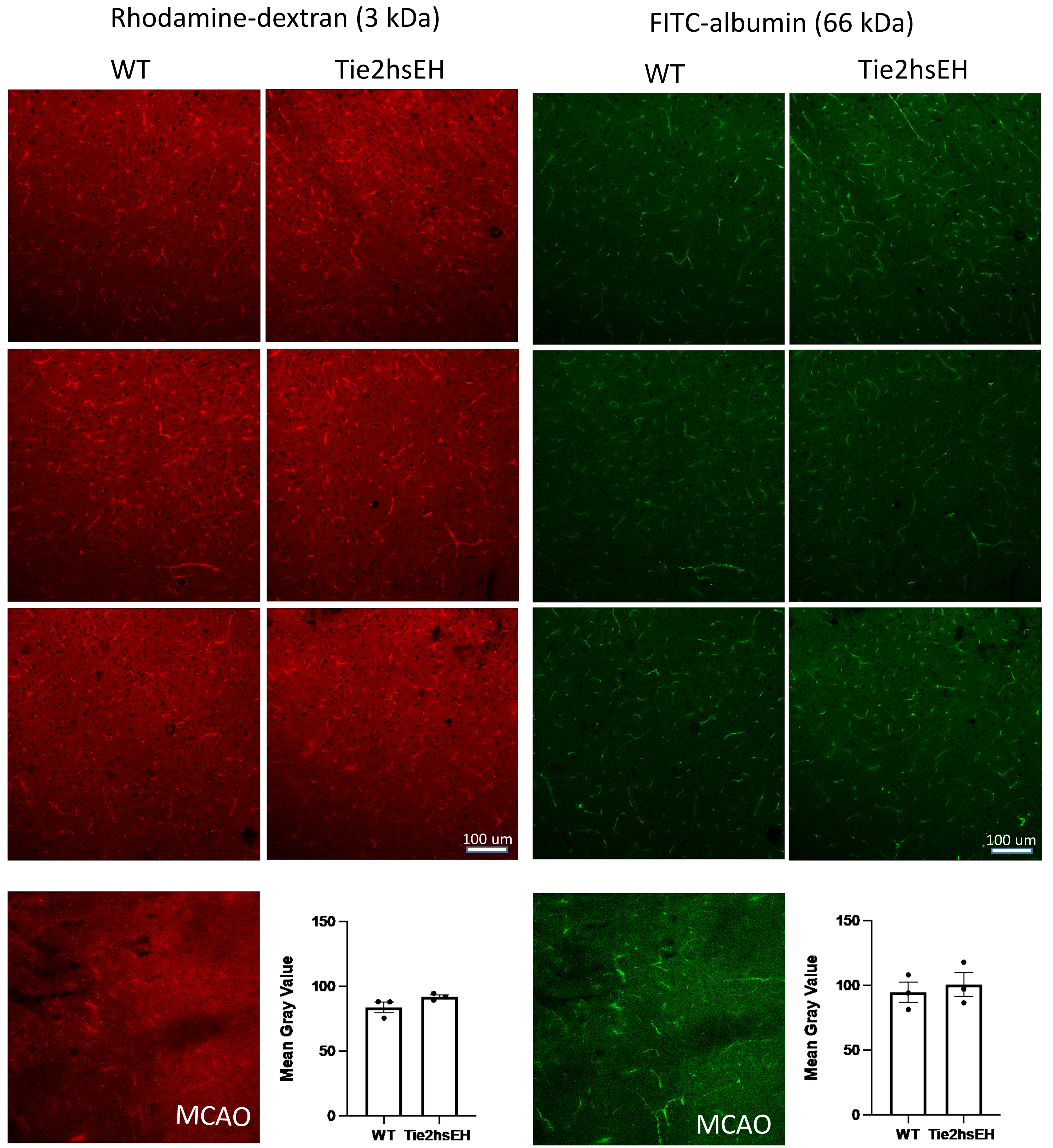

Supplementary Figure 6. Analysis of BBB permeability in WT and Tie2hsEH mice. 8.5-month-old mice (± 2 weeks) were used to determine BBB permeability to rhodamine-dextran (3 kDa; red) and FITC-albumin (66 kDa). Images from both WT and Tie2hsEH mice show dye confined within defined cerebral microvessels. The positive control (WT MCAO) shows some FITC-albumin within vessels, however most signal of both dyes is diffuse throughout the section indicating BBB permeability. Representative images from cortex, scale bar, 100μm. No significant differences were observed between groups, two-tailed t-test, n=3/group, data are represented as mean ± SEM.
